# Bridging the gut and mind: a narrative review of depression in Crohn’s disease and emerging gut-brain axis therapies

**DOI:** 10.3389/fnins.2026.1920926

**Published:** 2026-08-24

**Authors:** Zoë Pipa, Newton Howard

**Affiliations:** Department of Pharmacology & Physiology, Georgetown University, Washington, DC, United States

**Keywords:** Crohn’s disease, depression, gut-brain axis, inflammation, ketamine, psilocybin, SSRIs, vagus nerve stimulation

## Abstract

Depression is a highly prevalent and clinically significant comorbidity in Crohn’s disease (CD), a form of inflammatory bowel disease (IBD), and is associated with increased disease activity, hospitalization, and a reduced quality of life (QoL). While traditionally attributed to the psychosocial burden of chronic illness, emerging evidence indicates that depressive symptoms in CD arise from dysregulation of the gut-brain axis (GBA), a bidirectional network linking immune, microbial, and central nervous system (CNS) processes. Chronic intestinal inflammation, characterized by elevated cytokines such as tumor necrosis factor (TNF-α) and interleukin-6 (IL-6), may influence brain function through vagal signaling, hypothalamic-pituitary-adrenal (HPA) axis activation, and neuroimmune pathways. Concurrently, epithelial barrier dysfunction and microbial dysbiosis promote translocation of bacterial products, further amplifying systemic inflammation and neuroimmune signaling. This review consolidates preclinical and clinical evidence linking immune activation, microbial alterations, and neuroimmune signaling to depression in CD. The review compares emerging therapeutic strategies, such as ketamine enantiomers, vagus nerve stimulation, and microbiota-targeting natural compounds, with established therapies like serotonergic antidepressants, cognitive behavioral therapy, and biologic immunotherapies. Across these modalities, therapeutic effects on mood are heterogeneous and often dissociated from improvements in intestinal inflammation, highlighting the multifunctional nature of GBA dysfunction. Overall, these findings suggest that therapeutic responses in CD-associated depression may depend on the extent to which interventions modulate distinct components of the GBA, including immune, neural, and microbial pathways. These observations emphasize the need for integrative treatment strategies that address the multiple contributors of GBA dysbiosis. Future studies incorporating standardized and emerging psychiatric, immunological, and microbiome outcomes will be critical to identifying mechanisms for specific treatment responses in this population.

## 1 Introduction

Depression is a common and clinically significant comorbidity in Crohn’s disease (CD), a form of inflammatory bowel disease (IBD), affecting approximately 25%–32% of patients and contributing to increased disease activity, hospitalization, and reduced quality of life (QoL) (Lichtenstein et al., 2018; Veauthier and Hornecker, 2018). While depressive symptoms in CD patients have been historically attributed to the psychosocial burden of chronic illness, emerging evidence suggests that depression predates the onset of IBD, postulating a bidirectional and mechanistic relationship between the brain and gut (Ananthakrishnan et al., 2013; Pennazio et al., 2015). These findings support the conceptual framework that depression in CD may arise from underlying neuroimmune dysregulation rather than solely due to the psychosocial consequences of this disease.

The gut-brain axis (GBA) provides the biological framework for this interaction. The GBA is a bidirectional communication network linking the central nervous system (CNS), enteric nervous system (ENS), autonomic nervous system (ANS), immune system, and gut microbiota (Veauthier and Hornecker, 2018). Within this network, neural, endocrine, and immune pathways–including vagal afferent signaling and activation of the hypothalamic-pituitary (HPA) axis–coordinate communication between intestinal and central processes (Zanoli et al., 2020). In CD, chronic intestinal inflammation, characterized by elevated cytokines such as tumor necrosis factor-alpha (TNF-α), and interferon-gamma (IFN-γ), may extend beyond the gastrointestinal tract by promoting systemic inflammation and neuroimmune signaling; thereby contributing to depressive symptomatology (Chivite et al., 2021; Frolkis et al., 2018; Fuller-Thomson and Sulman, 2006; Hatamnejad et al., 2022; Jayasooriya et al., 2022; Weston et al., 2024; Zanoli et al., 2020).

Despite the increasing recognition of these interconnected pathways, therapeutic approaches to CD-associated depression remain heterogeneous. Biologic therapies effectively reduce intestinal inflammation but demonstrate inconsistent effects on depressive symptoms, while antidepressants and behavioral interventions improve mood outcomes with only variable impact on gastrointestinal disease activity (Dubinsky et al., 2021; Gracie et al., 2018; Hou et al., 2022; Späth-Schwalbe et al., 1998). Population-level studies demonstrated high rates of antidepressant usage in IBD, alongside substantial early discontinuation and suboptimal treatment duration, particularly among younger patients (Jayasooriya et al., 2022). These findings, together with evidence that pre-existing depression worsens disease severity, highlight limitations in current management strategies and the need for more targeted approaches to CD-associated depression (Ananthakrishnan et al., 2013; Flint et al., 2012; Frolkis et al., 2018; Lichtenstein et al., 2018; Mikocka-Walus et al., 2016). Emerging therapies, including ketamine, vagus nerve stimulation (VNS), and microbiota-targeting compounds, further highlight the complexity of targeting the GBA, as their effects may selectively influence neural, immune, or microbial components (Côté et al., 2003; Kumar et al., 2020; Petersson et al., 2011).

This review will synthesize current evidence linking neuroimmune, microbial, and epithelial mechanisms to depression in CD and comparatively evaluate therapeutic strategies targeting the gut-brain axis. By examining how differing interventions interact with immune, neural, and microbial pathways, this review aims to clarify the heterogeneity of therapeutic responses and inform the development of more integrative treatment strategies.

Because this review focuses specifically on Crohn’s disease, CD-specific evidence is emphasized whenever available. However, where mechanistic or therapeutic evidence from CD is limited, findings from IBD populations, ulcerative colitis (UC), or relevant preclinical studies are discussed to provide context and are identified accordingly throughout the manuscript.

## 2 The gut-brain axis

The GBA is a bidirectional communication network linking the CNS, ENS, ANS, immune system, and gut microbiota. Within this system, neural, endocrine, and immune pathways coordinate communication within the gut and brain, with key contributions from vagal afferent signaling and sympathetic nervous system activity. The ANS regulates GI functions, including motility, secretion, and mucus production, while simultaneously integrating visceral sensory input for central processing (Cussotto et al., 2019; Späth-Schwalbe et al., 1998; Veauthier and Hornecker, 2018). Through these interactions, the GBA serves as a critical interface through which intestinal physiology can influence central neuroimmune signaling.

### 2.1 Microbiota and the mucosal barrier

The gut microbiota are distributed along gradients from the proximal to distal GI tract and from the epithelial lining toward the lumen, where density increases. These microorganisms play a central role in maintaining the mucosal barrier, which protects epithelial cells against mechanical, chemical, and microbial pathogens while permitting selective diffusion of water, nutrients, and gases (Paone and Cani, 2020). The components of mucus are vast and consist of various species that contribute to the successful mucus turnover within the GI (Table 1). By creating a coat covering intestinal cells, it protects them from harmful substances, digestive enzymes, and bacteria. They also contribute to a diffusion barrier in which small molecules, such as water, ions, nutrients, and gases, can readily move toward enterocytes (Graff et al., 2009).

**TABLE 1:** Notable gut microbiota and their relevant secretions.

Microbiota species/group	Mechanism of action	Functional relevance to gut-brain axis
*Akkermanisa muciniphila*	mucin-degrading enzymes, outer membrane protein Amuc_1100	Degrades mucus, stimulates mucus production, goblet cell proliferation, and SCFA release
*Bacteroides thetaiotaomicron*	Glycosidases for mucin glycans	Switches between using mucin glycans and dietary carbs; contributes to mucus turnover
*Bacteroides fragilis*	Glycosidases	Mucin degradation; associated with outer mucus layer metabolism
*Bifidobacterium bifidum/longum*	SCFAs for mucin glycan metabolism	Stimulate MUC2 and MUC3 expression; increase mucus thickness and barrier function
*Lactobacillus* spp.	SCFAs, adhesion proteins	Stimulate MUC2 and MUC3 expression; increase mucus thickness and barrier function
*Ruminococcus gnavus*/*torques*	Mucin-degrading enzymes	Associated with increased mucus degradation
*Clostridium* spp.	Variable (some pathogenic, some commensal)	Some species adhere to and modulate mucus
*Faecalibacterium prausnitzii*	Butyrate	Anti-inflammatory, supports gut barrier, and mucus integrity
*Eubacterium rectale*/*cylindroides*	SCFAs (butyrate)	Colon mucus specialists contribute to mucus homeostasis
Proteobacteria	Variable, associated with stress, mucus production	Enriched in inner mucus during disease; potentially disrupts the mucus barrier
Segmented filamentous bacteria (SFB)	Tight adhesion to epithelium	Modulate immune response; colonize the mucus surface in mice

Data presented in this table are summarized from references (Cussotto et al., 2019; Graff et al., 2009; Kumar et al., 2020).

**FIGURE 1:**
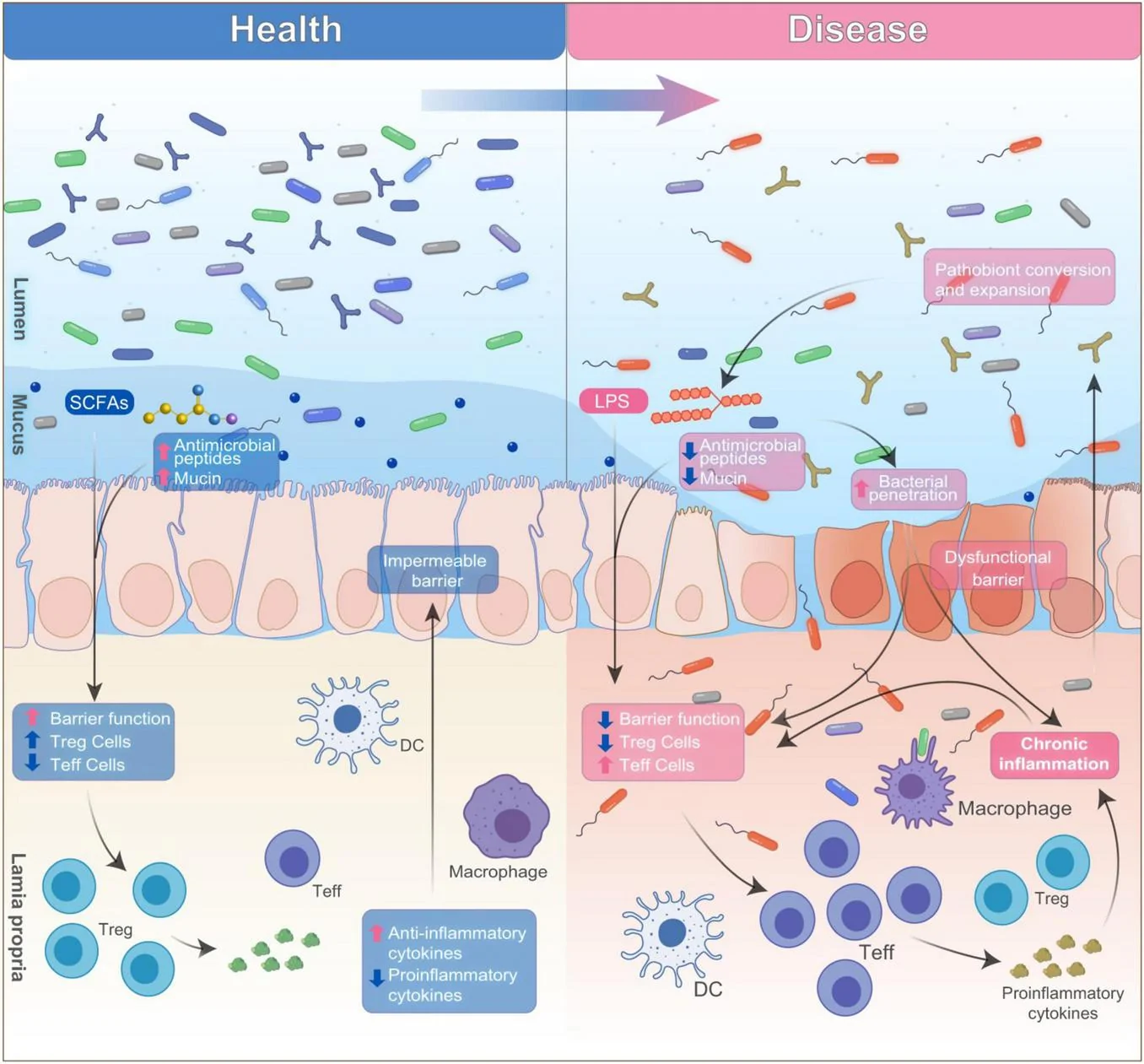
Factors affecting microbiota-associated chronic inflammation in healthy and disease states. Host genetics, diet, epithelial barrier integrity, immune signaling, and environmental exposures interact with gut microbial composition and function to regulate inflammatory tone. Dysregulation of these pathways contributes to chronic inflammation and systemic immune activation. Adapted from prior conceptual models (Hou et al., 2022).

In healthy conditions, the gut microbiota is diverse and functionally stable, supporting immune regulation and epithelial integrity. It is highly diverse, gene-rich, and varies by age, environment, and anatomical location. For example, *Enterobacteriaceae* predominate in the small intestine, whereas *Bacteroidaceae* and *Prevotellaceae* are more common in the colon. Microbiota diversity increases from childhood to adulthood and declines in older age, with reduced *Bifidobacterium* and increased *Clostridium* and *Proteobacteria* contributing to inflammation (Cussotto et al., 2019; Prechtl and Powley, 1990). Given the central role of gut microbiota in human health and disease, research has increasingly shifted from simply cataloging microbial composition to exploring functional interactions and causal mechanisms. Advances in high-throughput sequencing, computational modeling, and metabolomics are helping to elucidate how microbiota influences host physiology, offering new opportunities for microbiome-informed diagnostics and personalized therapeutic strategies. In Crohn’s disease (CD), dysbiosis is characterized by reduced microbial diversity and shifts in bacterial composition, including decreased beneficial species and increased pro-inflammatory taxa (Cussotto et al., 2019; Graff et al., 2009; Ozbey et al., 2025; Prechtl and Powley, 1990). These alterations impair barrier functions and promote immune activation (Figure 1).

A key mediator of microbiota-host interactions is the production of short-chain fatty acids (SCFAs), such as butyrate and acetate, which are generated through microbial fermentation of dietary substrates. SCFAs enhance mucosal integrity by stimulating MUC2 expression and mucus production, while also modulating immune responses and influencing enteric and central neural signaling pathways. SCFA signaling through receptors such as FFAR3 has been identified in the ENS and associated neural pathways, linking microbial metabolism to parasympathetic activity and central regulatory circuits involved in homeostatic and emotional processing (Graff et al., 2009).

In addition to SCFAs, gut microorganisms generate several bioactive metabolites that may influence gut-brain communication. Microbial metabolism of tryptophan produces indole derivatives that regulate epithelial barrier integrity, immune responses, and aryl hydrocarbon receptor (AhR) signaling. Similarly, primary bile acids are transformed by intestinal bacteria into secondary bile acids, which interact with receptors such as farnesoid X receptor (FXR) and Takeda G-protein-coupled receptor 5 (TGR5) to influence inflammatory signaling, metabolic homeostasis, and neuroendocrine function. Alterations in these metabolite networks have been reported in both inflammatory bowel disease and depressive disorders, suggesting a potential role in GBA dysregulation (Côté et al., 2003; Kumar et al., 2020; Santos et al., 1999).

Disruption of the mucosal barrier integrity initiates a cascade of gut-brain axis dysregulation. Increased intestinal permeability facilitates the translocation of microbial products, including lipopolysaccharides (LPS), into systemic circulation, promoting inflammatory signaling and neuroimmune activation (Emge et al., 2016; Graff et al., 2009; Matteoli and Boeckxstaens, 2013; Prechtl and Powley, 1990). Experimental models support this relationship, as dextran sodium sulfate (DSS)-induced colitis is associated with reduced mucus layer thickness and increased disease activity, highlighting the functional importance of barrier integrity in limiting inflammation (Petersson et al., 2011). Similarly, germ-free models exhibit markedly reduced mucus thickness, which can be restored following exposure to microbial products such as LPS, further demonstrating the role of microbiota in regulating mucosal structure (Emge et al., 2016; Prechtl and Powley, 1990).

These barrier disruptions promote activation of antigen-presenting cells (APCs) and downstream T-cell signaling pathways, leading to the release of pro-inflammatory cytokines, including interleukin-6 (IL-6) and TNF-α, which contribute to epithelial alterations, mucus dysregulation, and microbial imbalance. This sustained inflammatory signaling is associated with alterations in autonomic balance, including increased sympathetic nervous system activity and a reduction in vagal tone. Collectively, these processes reinforce dysbiosis and barrier dysfunction while contributing to systemic inflammation and neuroimmune signaling pathways implicated in anxiety, depressive symptoms, and reduced quality of life in IBD patients (Späth-Schwalbe et al., 1998; Veauthier and Hornecker, 2018).

In CD, chronic inflammation is also associated with the development of mesenteric adipose tissue expansion, or “creeping fat,” which is hypothesized to arise from increased intestinal permeability and translocation of microbial products into mesenteric tissue. This translocation promotes the activation of innate immune pathways, including antigen-presenting cells and cytokine signaling cascades (e.g., TNF-α, IL-6), which drive sustained local inflammation and adipose tissue remodeling (Graff et al., 2009; Späth-Schwalbe et al., 1998; Veauthier and Hornecker, 2018). While this process may function to contain microbial spread, it also leads to fibrotic tissue development and persistent immune overactivation.

Collectively, microbiota dysbiosis, impaired barrier integrity, and microbial translocation accumulate to promote systemic inflammation and cytokine signaling, which can influence CNS function through neuroimmune pathways, including the activation of the HPA axis and vagal afferent signaling, further contributing to depressive symptoms in CD (Chivite et al., 2021; Frolkis et al., 2018; Zanoli et al., 2020).

### 2.2 Serotonin signaling

A crucial mechanism within the GBA is its capacity to produce serotonin (5-HT). While serotonin is widely associated with CNS function, enterochromaffin cells (ECs) and the myenteric nerve plexus are primarily responsible for its production within the gastrointestinal tract (Bercik et al., 2011). Serotonin is synthesized from L-tryptophan via tryptophan hydroxylase (TPH1), producing 5-hydroxytryptophan (5-HTP), which is converted to 5-HT (Bercik et al., 2011). Following its release from ECs, 5-HT contributes to gastrointestinal motility, secretion, and sensory signaling through multiple serotonin receptor subtypes. Serotonin availability is additionally regulated through cellular uptake and subsequent metabolism by monoamine oxidases (Gomollón et al., 2017).

In addition to serving as a precursor for serotonin synthesis, tryptophan can be metabolized through the kynurenine pathway, a major route of tryptophan degradation that is influenced via inflammatory signaling. Pro-inflammatory cytokines, including interferon-gamma (IFN-γ), TNF-α, and IL-6, induce the activity of indoleamine-2,3-dioxygenase (IDO), the rate-limiting enzyme of kynurenine metabolism. Increasing IDO activity directs tryptophan away from serotonin production and toward downstream generation of kynurenine and its metabolites, including quinolinic acid and kynurenic acid, which influence glutamatergic neurotransmission, neuroplasticity, and neuroimmune signaling. Therefore, it is believed that chronic inflammation in Crohn’s disease may influence mood not only through cytokine-mediated neuroimmune signaling, but also alterations in tryptophan metabolism that affect both serotonergic and kynurenine pathways (Santos et al., 1999).

While serotonin signaling represents another important mechanism linking the gut and brain, peripheral serotonin itself does not cross the blood-brain barrier (BBB). Instead, gut-derived serotonin influences central processes indirectly through neuroimmune and neural pathways, including modulation of vagal afferent signaling and interactions with immune cells (Kakazu et al., 1999; Kalliolias and Ivashkiv, 2016). Through these pathways, alterations in intestinal serotonin signaling may impact CNS function and contribute to mood regulation.

Given the importance of serotonin in both gastrointestinal and central nervous system function, selective serotonin reuptake inhibitors (SSRIs) have been widely utilized to treat depressive symptoms. SSRIs prolong serotonin availability through inhibition of the serotonin transporter (SERT), influencing both central neurotransmission and peripheral gastrointestinal signaling. In addition to their antidepressant effects, SSRIs have been shown to alter gut motility, microbial composition, and vagal activity, further supporting their role as modulators of gut-brain axis function (Corridoni et al., 2014; Kakazu et al., 1999; Kalliolias and Ivashkiv, 2016).

### 2.3 The vagus nerve and stress pathways

The vagus nerve and stress-response systems are central regulators of gut-brain axis communication, integrating neural, endocrine, and immune signaling pathways. Psychological stress activates the hypothalamic-pituitary-adrenal (HPA) axis, leading to increased release of corticotropin-releasing hormone and glucocorticoids, which have been shown to disrupt intestinal epithelial integrity and increase permeability in experimental models (Imam et al., 2018). This stress-induced barrier dysfunction promotes microbial translocation and downstream immune activation.

Systemic inflammatory mediators can also influence immune cells within the CNS, known as microglia. Under inflammatory conditions, circulating cytokines and microbial products can promote microglial activation, resulting in a release of pro-inflammatory mediators including TNF-α, IL-1β, and reactive oxygen species (ROS). Persistent activation of microglia has been associated with impairments in neuroplasticity, alterations of neurotransmitter signaling, and depressive symptoms, suggesting additional mechanisms through which chronic intestinal inflammation may influence both mood and behavior (Medina-Rodriguez and Beurel, 2022; Menard et al., 2017).

Chronic systemic inflammation may additionally alter blood-brain barrier (BBB) integrity. Experimental studies demonstrate that pro-inflammatory cytokines, including TNF-α and IL-6, can increase BBB permeability, facilitating immune-to-brain communication and the entry of inflammatory mediators into the central nervous system. Increased BBB permeability may therefore amplify neuroinflammatory signaling and contribute to alterations in mood, cognition, and stress responsiveness observed in inflammatory disorders. (Liang et al., 2025; Medina-Rodriguez and Beurel, 2022; Varatharaj and Galea, 2017).

In parallel, stress is associated with increased sympathetic nervous system activity and elevated catecholamine release, including epinephrine and norepinephrine. These neuroendocrine signals stimulate immune cells such as mast cells and macrophages, promoting the release of pro-inflammatory cytokines and amplifying intestinal inflammation (Mantovani et al., 2011). This establishes a feed-forward loop in which stress-induced neuroendocrine signaling exacerbates barrier dysfunction, immune activation, and microbial imbalance.

Conversely, the vagus nerve exerts anti-inflammatory effects through the cholinergic anti-inflammatory pathway, in which efferent vagal signaling suppresses cytokine production via nicotinic acetylcholine receptor-mediated mechanisms (Berlin et al., 1993; Feagan et al., 2013; Solomon et al., 2019). Reduced vagal tone during stress diminishes this protective pathway, allowing pro-inflammatory signaling to predominate. Experimental studies further demonstrate that vagal signaling plays a key role in microbiota-gut-brain communication, as modulation of gut microbiota through probiotic interventions alters behavior in a vagus-dependent manner (D’Onofrio et al., 2025; Stevens et al., 2017).

Although the precise mechanisms underlying visceral hypersensitivity in IBD remain incompletely defined, current evidence suggests that increased intestinal permeability facilitates translocation of microbial products such as lipopolysaccharides (LPS), which activate innate immune pathways and promote the release of pro-inflammatory cytokines. These cytokines influence central nervous system function through vagal afferent signaling and HPA axis activation, while also sensitizing visceral pain pathways, including spinothalamic and limbic circuits involved in emotional processing. Collectively, these findings support the hypothesis that immune activation, microbial dysbiosis, epithelial barrier dysfunction, and altered neuroimmune signaling contribute to gut-brain axis dysregulation in Crohn’s disease (Ananthakrishnan et al., 2013; Corridoni et al., 2014; Solomon et al., 2019). However, many of these relationships remain associative rather than causally established in human populations. Therefore, current gut-brain axis models should be interpreted as biologically plausible frameworks which still require experimental validation.

**FIGURE 2:**
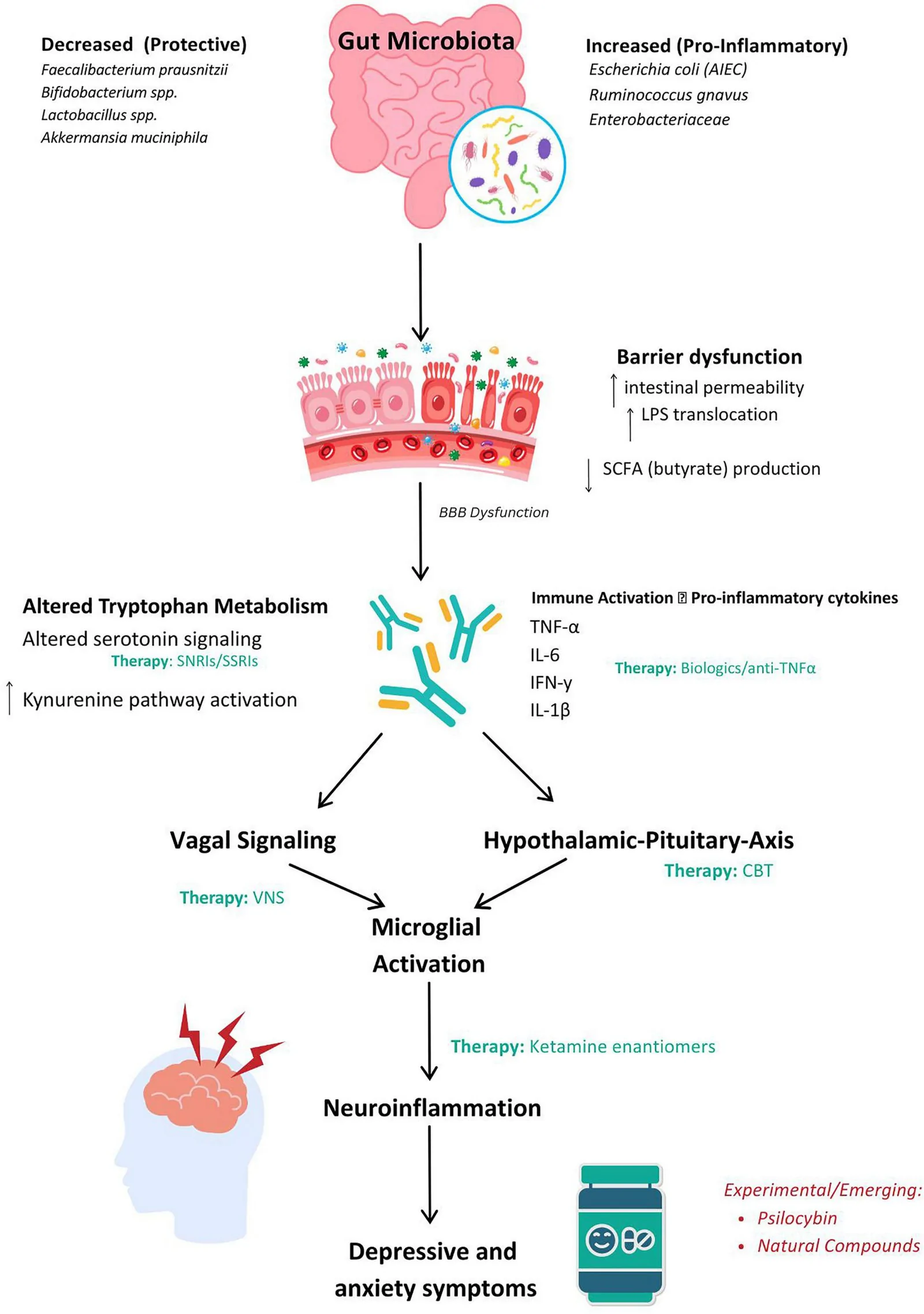
Proposed mechanisms linking Crohn’s disease to depression through the gut-brain axis. Gut microbial dysbiosis in CD is associated with impaired epithelial barrier integrity, increased intestinal permeability and microbial translocation, reduced production of protective microbial metabolites such as short-chain fatty acids (SCFAs), altered tryptophan metabolism, and activation of pro-inflammatory immune pathways. Systemic inflammatory signaling may subsequently influence central processes through vagal signaling, hypothalamic-pituitary-adrenal (HPA) axis activity, blood-brain barrier dysfunction, and microglial activation, contributing to neuroinflammatory pathways implicated in depressive symptoms. These pathways are interconnected and bidirectional and should not be interpreted as a strictly sequential or causally established pathway in humans. Created by the authors.

Beyond neuroinflammation, chronic inflammatory and stress-related signaling may affect neural circuits involved in mood regulation. Brain regions including the prefrontal cortex, hippocampus, and amygdala are implicated in depression and are sensitive to alterations in inflammatory signaling, HPA-axis activity, and neuroplasticity. Pro-inflammatory cytokine signaling and microglial activation may contribute to impaired synaptic plasticity and altered neurotransmitter function within these regions, while changes in neurotrophic factors such as BDNF may further influence neuronal resilience and mood regulation. Although these mechanisms provide plausible central neural pathways linking systemic inflammation to depressive symptoms, their specific contribution to depression in CD remains incompletely characterized. Collectively, these microbial, epithelial, immune, metabolic, neuroendocrine, and neural pathways provide an integrated mechanistic framework through which intestinal inflammation may contribute to depressive symptoms in CD (Figure 2).

## 3 Search strategy

This narrative review was informed by a structured literature search conducted using PubMed, Google Scholar, and ScienceDirect to identify studies examining the relationship between Crohn’s disease (CD), depression, the gut-brain axis (GBA), and emerging therapeutic strategies. The search included articles published in English between January 1980 and June 2026, reflecting both foundational discoveries in gut-brain signaling and recent advances in neuroimmune and microbiome research.

Searches were performed using combinations of Medical Subject Heading (MeSH) terms and free-text keywords, including “Crohn’s disease,” “inflammatory bowel disease,” “depression,” “gut-brain axis,” “gut microbiota,” “microbiome,” “intestinal permeability,” “blood-brain barrier,” “microglia,” “vagus nerve,” “hypothalamic-pituitary-adrenal axis,” “serotonin,” “kynurenine,” “short-chain fatty acids,” “biologics,” “anti-TNF,” “selective serotonin reuptake inhibitors (SSRIs),” “ketamine,” “vagus nerve stimulation,” “psilocybin,” “rosemary,” “jasmine tea,” and “cognitive behavioral therapy.” Boolean operators (AND, OR) were used to refine searches and identify studies addressing multiple components of the gut-brain axis utilizing single or combination therapies (Table 2).

**TABLE 2:** Literature search strategy and Boolean search terms.

Database	Search string
PubMed	(Crohn’s disease OR inflammatory bowel disease) AND (depression OR anxiety OR depressive symptoms OR Major Depressive Disorder) AND (gut-brain axis OR GBA OR microbiome OR gut microbiota)
PubMed	(Crohn’s disease OR inflammatory bowel disease OR ulcerative colitis) AND (depression OR anxiety) AND (biologics OR anti-TNF OR SSRI OR ketamine OR vagus nerve stimulation OR cognitive behavioral therapy)
PubMed	(Crohn’s disease OR inflammatory bowel disease) AND (microglia OR blood-brain barrier OR kynurenine OR serotonin OR short-chain fatty acids OR indoles OR bile acids)
Google Scholar	(Crohn’s disease OR inflammatory bowel disease OR ulcerative colitis) AND (depression OR anxiety) AND (biologics OR anti-TNF OR SSRI OR ketamine OR vagus nerve stimulation OR cognitive behavioral therapy OR natural compounds OR depressive therapies)
ScienceDirect	(Crohn’s disease OR inflammatory bowel disease OR ulcerative colitis) AND (depression OR anxiety) AND (biologics OR anti-TNF OR SSRI OR ketamine OR vagus nerve stimulation OR cognitive behavioral therapy OR natural compounds OR depressive therapies)

Studies were considered for inclusion if they met the following criteria: (1) investigated Crohn’s disease or inflammatory bowel disease with clearly identified disease subtype when available, (2) evaluated depression, anxiety, or gut-brain axis mechanisms relevant to neuropsychiatric outcomes, (3) examined established or emerging therapeutic interventions, or (4) provided mechanistic insight through clinical, translational, or preclinical research. Randomized controlled trials, cohort studies, case-control studies, systematic reviews, meta-analyses, and high-quality experimental studies were prioritized.

Studies were excluded if they focused on gastrointestinal disorders unrelated to inflammatory bowel disease, lacked relevance to depression or gut-brain axis biology, were not published in English, or consisted solely of conference abstracts, editorials, or opinion articles without original data or substantial scientific synthesis.

Titles and abstracts were initially screened for relevance, followed by full-text review of eligible publications. Reference lists of key studies and recent review articles were also manually searched to identify additional relevant publications that were not captured during the initial database search.

Because this manuscript is presented as a narrative review, studies were selected based on their scientific relevance and contribution to understanding the relationship between Crohn’s disease, depression, and the gut-brain axis rather than through formal systematic review methodology. Greater emphasis was placed on studies with robust experimental design, clearly characterized patient populations, and mechanistic findings that advanced current understanding of gut-brain axis dysfunction. The final narrative drew on 94 references, encompassing clinical investigations, translational research, and preclinical models evaluating immune, microbial, neural, and therapeutic mechanisms relevant to CD-associated depression.

## 4 Current therapeutic approaches to depression in Crohn’s disease

### 4.1 Immunotherapies

This section primarily discusses clinical studies in patients with Crohn’s disease (CD), supplemented by mixed IBD cohorts when CD-specific evidence is limited. Since inflammation, disease activity, sleep disturbances, and psychological stressors together can impact IBD symptoms, biologic therapies may indirectly influence mood through anti-inflammatory pathways (Stevens et al., 2017). Anti-tumor necrosis factor (anti-TNF) agents have revolutionized the treatment of CD and ulcerative colitis (UC). However, because many clinical investigations evaluate mixed IBD cohorts, distinctions between CD-specific and broader IBD findings are noted below where applicable. Anti-TNF agents are prescribed to diminish the need for steroid therapy, and their effects have been shown to promote mucosal healing and reduce hospitalization time, leading to dramatic improvements in QoL scores among IBD patients (Di Petrillo et al., 2025). However, other clinical studies in patients with IBD have shown that not every patient responds positively to anti-TNF agents; as one-third see no improvements even after utilizing a second or third treatment (Stidham et al., 2014). Few studies have evaluated the effectiveness of a second anti-TNF agent in IBD patients who did not achieve remission with their first anti-TNF drug (Gisbert and Chaparro, 2020).

Tumor necrosis factor-alpha is a key pro-inflammatory cytokine involved in the pathogenesis of Crohn’s disease. Through activation of TNF receptors and downstream inflammatory signaling pathways, TNF-α promotes leukocyte recruitment, cytokine production, and mucosal injury. Consequently, anti-TNF therapies reduce intestinal inflammation and have become a cornerstone of modern IBD management (Sjöstedt et al., 2021).

Within CD individuals, overactive T-helper 1 (Th1) cells, a central component of the immunoinflammatory process in CD, produce stimulatory cytokines interferon-gamma (IFN-γ), IL-2, IL-12, and TNF-α and recruit other cells in the pathological reaction. The role of the Th1 cells in the pathogenesis of CD arises from several pathophysiological changes: The production of granulomas, which are a characteristic pathological finding in CD, is dominated by Th1 cells (ten Hove et al., 2002). The production of Th1-type cytokines increases during CD relapses and remissions (Agnholt et al., 2004). Th1 cells also mediate experimental colitis in several models, but they have also been shown to induce intestinal inflammation when transferred from one animal to another (Bektaş et al., 2020). A prime component of Th1-mediated colitis is the activation of TNF-α.

Early clinical studies demonstrated that infliximab reduces inflammatory activity in IBD, including alterations in circulating leukocytes and neutrophil populations and intestinal inflammatory responses (D’Onofrio et al., 2025). Separately, studies examining TNF-α inhibition in patients with depression have suggested that antidepressant responses may be more apparent among individuals with elevated baseline inflammatory biomarkers such as CRP (Yoo et al., 2020). These findings raise the possibility that baseline inflammatory status may influence psychiatric response to anti-inflammatory treatment; however, evidence from non-IBD psychiatric populations should not be directly extrapolated to CD-associated depression. Within *in-vitro* cultures of intestinal T-lymphocytes from CD patients, infliximab was shown to suppress the production of IFN-γ and TNF-α compared to cultures of healthy controls (Bektaş et al., 2020). Complementary *in vivo* studies demonstrated that infliximab induced apoptosis of activated lamina propria T cells, resulting in reduced mucosal T-cell density in colonic biopsies within 24 h of infusion (ten Hove et al., 2002). Beyond these changes, infliximab has also been shown to inhibit GM-CSF (granulocyte-macrophage colony-stimulating factor) production by mucosal T cells, most of which are identified as TCR α/ß + CD4 + CD45RO+ memory cells. Patient biopsies collected after infliximab treatment showed a significant reduction in GM-CSF secretion by harvested mucosal lymphocytes, and additional *in vitro* stimulation experiments further confirmed infliximab’s GM-CSF inhibitory activity (Agnholt et al., 2004; Bektaş et al., 2020).

Additional immune mediators, including GM-CSF, have also been implicated in CD pathogenesis and correlate with disease activity, further illustrating the complexity of inflammatory signaling networks involved in intestinal disease progression (Hanna-Jairala and Drossman, 2024).

Clinical studies of mixed IBD cohorts have shown that intestinal inflammation is characterized by increased leukocyte infiltration of the intestinal mucosa; therefore, blocking leukocyte transport is a crucial strategy for treating the inflammatory response of IBD (Cussotto et al., 2019). Liang et al. (2025) identified prevalent immune cells in IBD patients, indicating that high levels of neutrophils were positively associated with both CD and ulcerative colitis (UC) prevalence. As neutrophils eliminate microorganisms through phagocytosis and degranulation, there is a release of reactive oxygen species (ROS) and neutrophil extracellular traps (NETs). This excessive release of ROS induces an overwhelming amount of GI oxidative stress, furthering inflammation in the intestinal mucosa by activating redox-sensitive pathways and transcription factors (Biasi et al., 2013; Mantovani et al., 2011).

Other therapies explored include Vedolizumab, a gut-selective monoclonal antibody that targets the α4β7 integrin expressed on circulating lymphocytes, preventing interactions with mucosal addressin cell adhesion molecule-1 (MAdCAM-1) on intestinal endothelial cells. This blockage inhibits lymphocyte trafficking within the gut mucosa, reducing overall intestinal inflammation while simultaneously sparing systemic immune suppression. As a result, vedolizumab differs mechanistically from systemic anti-TNF-α therapies and may provide distinguishing effects on extraintestinal and neuropsychiatric outcomes (Berlin et al., 1993; Feagan et al., 2013; Solomon et al., 2019).

Stevens et al. (2017) conducted a study that enrolled both patients with CD and UC, and its findings should be interpreted as evidence from a mixed IBD population rather than CD alone. Within the study, they tested the efficacy of using Vedolizumab to treat poor sleep quality, anxiety, and depression in IBD patients. They compared the effectiveness of an anti-TNF-α cohort (adalimumab, infliximab, certolizumab, or golimumab) in patients >18 years old who had a confirmed CD or UC diagnosis, and were initiating one of the therapies listed as part of routine care. They aimed to understand which therapy would best reduce sleep disturbances, as these disturbances contribute greatly to the elevation of CRP, IL-1β, IL-6, and TNF-α levels. Both patients and providers observed early and sustained improvements in sleep quality and mood in both gut-selective anti-integrin and systemic anti-TNF-α therapy groups, leading to the hypothesis that a reduction in disease burden and circulating inflammation, rather than just to the GI tract, could contribute to improving both depression and sleep quality. These findings remain inconclusive, as studies do not confirm if anti-inflammatory properties directly impacted the neurochemical influence on mood, or if they reduced the susceptibility to mood-impacting behaviors, such as poor sleep.

D’Onofrio et al. (2025) created a pilot study to monitor the psychopathological profile of biological agent treatment for IBD patients to understand the association between the administration of these agents and psychopathology. Through a naturalistic prospective study, patients with mixed IBD were assigned to either a biological agent group (incl. tofacitinib) or conventional therapy. They completed Hamilton Depression Rating Scales (HAM-D) and Hamilton Anxiety Rating Scales (HAM-A), Young Mania Rating Scale (YMRS), and Brief Psychiatric Rating Scale (BPRS), and disease activity [Harvey-Bradshaw Index (HBI) and Mayo Score] at baseline. Patients on biological drugs who completed the three months of treatment (*N* = 32) and six months of treatment (*N* = 20) scored lower on the Mayo compared to baseline. Patients on conventional treatments obtained significant drops from baseline on the HBI after one and three months of treatment (*N* = 30) and at the 6-month endpoint (*N* = 11). Both groups showed no improvement or worsening on the psychiatric rating scale, indicating no evidence of worsening mood. It remains unclear whether improvements in depressive symptoms following anti-inflammatory treatment with agents such as infliximab reflect direct effects on neuroimmune pathways or occur indirectly through reductions in intestinal inflammation and overall disease burden. Further research is needed to determine whether these therapies have independent antidepressant effects or provide greater benefit when combined with conventional antidepressant treatments.

Overall, these findings suggest that biologic therapies may improve depressive symptoms in some patients with CD; however, their antidepressant effects appear heterogeneous and may depend, in part, on baseline inflammatory activity. Anti-TNF-α agents such as infliximab have been associated with improvements in mood among patients with elevated inflammatory biomarkers, supporting the hypothesis that reductions in systemic inflammation may contribute to improved psychiatric outcomes (D’Onofrio et al., 2025). Studies of other biologics, including vedolizumab, suggest that improvements with depression, anxiety, sleep quality, and QoL may occur alongside reductions in disease activity and symptom burden, making it difficult to distinguish direct neuroimmune effects from the psychological benefits of improved physical health (Stevens et al., 2017). Moreover, findings across studies are inconsistent regarding the relationship between changes in disease activity and psychiatric outcomes (D’Onofrio et al., 2025). Interpretation is further limited by differences in biologic class, baseline inflammatory activity, study populations, psychiatric outcome measures, and study design. Therefore, biologic therapies should not be considered independent antidepressant treatment for CD-associated depression. Instead, they may reduce inflammation-associated contributors to depressive symptoms in specific patient cohorts, while persistent psychiatric symptoms may require additional psychological or pharmacological treatment (D’Onofrio et al., 2025; Sanghavi et al., 2022; Wang et al., 2023; Yoo et al., 2020).

### 4.2 SSRIs

Unlike immunotherapies, SSRIs do not directly target intestinal inflammation, but are prescribed to alleviate comorbid depression and anxiety through central serotonergic modulation. SSRIs remain the most commonly prescribed antidepressants in patients with CD and UC. Most available clinical evidence evaluating antidepressants includes mixed IBD populations, with relatively few studies focusing exclusively on CD. Early small observational studies speculated that SSRIs might confer benefits in CD by reducing the need for corticosteroids or anti-TNF-α therapies. However, larger and more recent analyses reveal more complex findings. Meta-analyses of survey-based studies indicate that SSRIs improve depressive or anxiety symptoms in only approximately 25% of IBD patients (Gisbert and Chaparro, 2020). Moreover, in a large national cohort, Jayasooriya et al. (2022) found that both SSRIs and tricyclic antidepressants (TCAs) were linked to greater dependence on corticosteroids and poorer long-term outcomes, suggesting that not all antidepressant classes are equally beneficial to this patient population.

Systematic reviews of randomized controlled trials (RCTs) provide a more balanced perspective. Weston et al. (2024) reviewed SSRIs and SNRIs in IBD patients and found that across the three RCTs, antidepressants significantly improved depressive symptoms and quality of life (QoL), with SSRIs showing a trend toward reduction of anxiety. SNRIs demonstrated a broader clinical efficacy, improving depressive symptoms, anxiety, and QoL simultaneously. Importantly, the tolerance of both SSRIs and SNRIs was comparable to placebo, aside from mild adverse effects such as nausea. Although the meta-analyses showed no statistical heterogeneity (I^2^ = 0%), indicating consistent effects across studies, interpretation is limited by the small number of trials, variability in antidepressant class, disease activity, and outcome measures. Beyond their central nervous system effects, SSRIs exert peripheral immunomodulatory and microbiome-related effects that may influence disease course in IBD, although much of this evidence remains preclinical or associative.

Clinical limitations arise from the gastrointestinal side effects of SSRIs. These medications accelerate intestinal motility, which can exacerbate nausea, diarrhea, and abdominal pain/discomfort, symptoms that already burden many CD patients. Furthermore, SSRIs may increase the risk of gastrointestinal bleeding, a potential concern in patients with IBD and mucosal ulcerations (Hatamnejad et al., 2022). Mechanistically, these effects are thought to arise from SSRI-mediated modulation of immune signaling pathways, including TLR4 activation in glial and peripheral immune cells. Their peripheral actions may contribute to gut dysbiosis by increasing both TNF-α and IL-1β cytokine release, contributing to an increase in circulating CD4+ and CD8+ T cells. Activated TLR4 increases available cyclic-AMP (cAMP) in microglia and macrophages, inhibits interferon-gamma (IFN-γ) production via Th-cells, and increases circulating IL-10 (Mikocka-Walus et al., 2016). These immune cell shifts may contribute to gut dysbiosis by altering microbial composition, reducing bacterial diversity, and disrupting efflux pump function (Sjöstedt et al., 2021). Some studies have also suggested SSRIs can indirectly disturb microbial balance through sleep disruption, as SSRI-related sleep apnea and intermittent hypoxia (IH) are linked in shifts with aerobic and anaerobic bacterial colonies. These shifts can cause an inhibition of key microbial efflux pumps. Notably, such effects are more pronounced in microbial-rich regions like the ascending colon, where SSRI concentration may be the highest (Hatamnejad et al., 2022).

Selective serotonin reuptake inhibitors exert pleiotropic effects across the central nervous system, gastrointestinal tract, and immune system, positioning them as central acting antidepressants that may also indirectly modulate peripheral components of the GBA. Centrally, SSRIs increase synaptic serotonin availability, improving mood and anxiety symptoms (Gracie et al., 2018; Späth-Schwalbe et al., 1998). Peripherally, serotonin signaling plays a critical role in gastrointestinal motility, epithelial secretion, and immune regulation, with 90% of serotonin being produced in the gut. However, it is important to note that gut-derived serotonin cannot cross the BBB, meaning that mood effects are indirectly mediated through vagal, immune, and microbial pathways rather than through neurotransmitter changes (Côté et al., 2003). In inflammatory conditions such as CD, altered serotonergic signaling may influence immune cell activation, epithelial barrier functionality, and host-microbiome interactions (Mikocka-Walus et al., 2016; Murrough et al., 2013). This peripheral-central connection may help explain why some SSRIs are effective in certain subsets of CD patients, but not others.

Given these therapeutic limitations, alternative antidepressant therapies should be brought to light. TCAs, while occasionally used in CD patients, are hindered by anticholinergic side effects and cardiovascular risks. SNRIs appear to be more effective across mood and anxiety symptoms but still cause negative side effects, such as nausea and GI intolerance. Bupropion has also been investigated as an alternative antidepressant in patients with IBD. In addition to improving depressive symptoms, bupropion slightly slows gut motility, reduces TNF-α levels, improves fatigue, and may have a more favorable sexual side-effect profile than serotonergic antidepressants; a key QoL factor that can be overlooked when analyzing chronic disease management (Fujita et al., 2021; Kishimoto et al., 2016). However, high-quality competitive trials of these agents in CD-specific populations remain limited.

In summary, although SSRIs remain the most widely prescribed antidepressants in IBD, their variable efficacy, gastrointestinal side effects, and potential impact on gut microbial and immune homeostasis make their therapeutic role in CD-associated depression controversial. Future RCTs comparing antidepressant classes in CD populations specifically, ensuring an integration of psychiatric, immunological, and gastrointestinal outcome measures, are needed to determine if SSRIs should remain as a first-line therapy, or be replaced by alternatives such as SNRIs, bupropion or beyond.

### 4.3 Psychotherapy/lifestyle interventions

Psychological interventions, such as cognitive behavioral therapy (CBT) and psychotherapy, have been explored to alleviate depression in Crohn’s disease (CD), given the bidirectional interactions between the gut and brain. Evidence evaluating CBT has largely been derived from mixed IBD cohorts rather than exclusively CD populations. For example, Wang et al. (2023) conducted a meta-analysis of randomized controlled trials (RCTs) published through October 2022 to evaluate the effects of CBT on quality of life (QoL) and mental health in IBD patients. QoL was assessed using the Inflammatory Bowel Disease Questionnaire (IBDQ), while anxiety and depressive symptoms were measured with the Hospital Anxiety and Depression Scales (HADS-A for anxiety, and HADS-D for depression). The analysis also considered treatment duration, follow-up length, and the specific CBT interventions employed.

Overall, CBT was associated with a significant reduction in anxiety symptoms (HADS-A scores) across studies (SMD: −0.39, 95% CI: [−0.67, −0.10], *p* = 0.008), although substantial heterogeneity was observed (I^2^= 76%). Following sensitivity analysis excluding one study contributing to heterogeneity, heterogeneity decreased (I^2^= 46%), and CBT remained associated with a significant reduction in anxiety compared with controls (SMD: −0.25, 95% CI: [−0.45, −0.05], *p* = 0.01). Pooled analysis of six intervention groups reporting HADS-D scores similarly demonstrated a significant reduction in depressive symptoms with CBT (SMD: −0.17, 95% CI: [−0.31, −0.02], *p* = 0.02), with no observed heterogeneity (I^2^= 0%). Longer-term CBT interventions (≥12 weeks) were associated with significant improvements in IBD-specific quality of life, although further research is needed to determine whether treatment duration similarly influences anxiety and depressive outcomes (Wang et al., 2023).

In a separate RCT, Hunt et al. (2020) compared self-help CBT to an active psychoeducational control. Both groups improved in catastrophizing, visceral sensitivity, and health-related quality of life (HRQoL), but the CBT group showed larger pre-post effect sizes. Importantly, only participants receiving CBT experienced significant and sustained improvements in depression and anxiety, maintained at 3-month follow-up (*p* < 0.05). Limitations included online recruitment without verified IBD diagnoses, untracked additional therapies, imperfect disease activity measures, and a high attrition rate (46%). Despite these factors, the study supports CBT as an effective intervention beyond psychoeducation, benefiting patients across varying baseline severity levels. Collectively, these findings support CBT as a potentially useful adjunctive intervention for psychological symptoms in IBD; however, evidence specific to CD remains limited, and whether these psychological improvements correspond to measurable changes in GBA remains unclear.

## 5 Emerging therapies for depression in Crohn’s disease

The therapeutic approaches discussed in this section vary substantially in their level of clinical development. Although some, such as ketamine, have established applications in psychiatric populations, evidence supporting their use specifically for depression in Crohn’s disease remains limited. Other approaches, including psilocybin and microbiota-targeting natural compounds, are supported predominantly by preclinical or experimental evidence. Accordingly, these therapies should be considered investigational in the context of CD-associated depression and are not currently supported for routine clinical use.

### 5.1 Ketamine

Clinical evidence evaluating ketamine specifically in CD remains limited, although extensive evidence exists in major depressive disorder (MDD). The antidepressant properties of intravenous ketamine for MDD were first demonstrated in a double-blind, placebo-controlled trial by Berman et al. (2000), and later replicated by Murrough et al. (2013), who used midazolam as an active control. These studies consistently showed that ketamine produced rapid improvements in depressive symptoms (Su et al., 2017). More recently, esketamine (S-ketamine), the FDA-approved intranasal enantiomer, has demonstrated efficacy in treatment-resistant depression (TRD) and other neuropsychiatric populations (Hashimoto, 2019).

Ketamine exists as two enantiomers, (S)-ketamine and (R)-ketamine, which differ in receptor affinity and biological effects. (S)-ketamine has been more extensively studied in humans and exhibits a higher affinity for N-methyl-D-aspartate (NMDA) receptors, contributing to rapid antidepressant effects. Preclinical studies demonstrate that (S)-ketamine enhances neuroplasticity by increasing levels of brain-derived neurotrophic factor (BDNF) and postsynaptic density protein 95 (PSD-95) in key brain regions, including the prefrontal cortex, hippocampus, and dentate gyrus. These changes restore dopaminergic activity and synaptic connectivity in rodent models of chronic stress and social defeat, highlighting its potential to reverse stress-induced neural impairments (Berthoud et al., 1991; Bonaz et al., 2017; Cailotto et al., 2014; Fleshner et al., 1995; Pavlov et al., 2003; Wang et al., 2023; Zhang et al., 2023).

By contrast, (R)-ketamine remains less explored clinically, but shows promising preclinical effects relevant to the gut-brain axis. In multiple sclerosis mouse models, (R)-ketamine promoted favorable gut microbiota composition changes, increasing the abundance of beneficial species such as *Lactobacillus intestinalis, Barnesiella visericola, Faecalibaculum rodentium*, and *Prevotella loescheii*. These microbes are associated with microbial stability and immunoregulation (Wang et al., 2022), suggesting that (R)-ketamine may influence mood indirectly through gut-brain interactions. While clinical studies in CD are lacking, these findings suggest that ketamine enantiomers may differentially influence neuroimmune and microbiome pathways; however, the relevance of these mechanisms to CD-associated depression remains speculative and requires clinical validation.

In the first double-blind RCT evaluating ketamine in CD patients, Zhang et al. (2023) randomized 124 patients with baseline Hamilton Depression Rating Scale-17 (HAMD-17) scores of 8–24 (24 = most severe) to receive either placebo [intravenous (IV) saline: 0.9% during anesthesia induction, followed by 50 mL 0.9% saline for more than 30 min after] or low-dose (S)-ketamine (continuous infusion at 0.12 mg/kg/h during anesthesia). Postoperatively, all patients received standard patient-controlled analgesia. Primary outcomes (HAMD-17) and secondary outcomes Patient Health Questionnaire-9-item (PHQ-9) and Quality of Recovery-15 scale (QoR-15) were assessed at days 1, 3, 7, and 30. Pain scores using the numerical rating scale (NRS), were also recorded on days 1, 2, and 7.

Based on a single RCT, patients receiving (S)-ketamine reported significantly lower depression and pain scores, as well as higher QoR scores at days 3 and 7, compared to controls. However, there were no differences in systemic inflammatory biomarkers (IL-6 and CRP) between groups. This suggests that while (S)-ketamine has antidepressant and analgesic benefits, these effects may not be mediated through anti-inflammatory pathways. A further limitation was the potential confounding influence of intraoperative dexmedetomidine, which may have interacted with (S)-ketamine’s effects. Given that current CD-specific evidence is largely limited to a single perioperative RCT, additional trials are needed to determine the durability, dose dependence, and enantiomer-specific effects of ketamine, and to establish whether these findings extend beyond the perioperative setting (Zhang et al., 2023).

### 5.2 Vagus nerve stimulation (VNS)

Within the gut-brain axis (GBA), the vagus nerve (VN) provides bi-directional communication between the gut and brain via the autonomic nervous system (ANS) (Cailotto et al., 2014; Wang et al., 2003). Approximately 80% of VN fibers are afferent, transmitting visceral, somatic, and taste signals, while the remaining 20% are efferent, regulating gastrointestinal motility and serotonin release from enterochromaffin cells (Cailotto et al., 2014). Preganglionic efferent fibers arise from the dorsal motor nucleus of the vagus (DMNV) and project to postganglionic enteric neurons, whereas vagal afferents project to the nucleus tractus solitarius (NTS) (Fleshner et al., 1995). Acetylcholine (ACh) serves as the primary neurotransmitter in these circuits, binding to nicotinic and muscarinic receptors on enteric neurons, which in turn modulate the gut immune response (Biasi et al., 2013; Mantovani et al., 2011; Rubio et al., 2014). Chronic inflammation of vagal pathways has been linked to hypothalamus-pituitary-adrenal axis (HPA) dysfunction and dysregulated amygdala-prefrontal cortex activity, both observed in IBD (Borovikova et al., 2000; Wang et al., 2003).

Two anti-inflammatory mechanisms of the VN have been discovered. First, pro-inflammatory cytokines such as interleukin-1β (IL-1β) activate vagal afferents, which signal the NTS and stimulate corticotropin-releasing factor (CRF) neurons in the hypothalamus. This cascade drives adrenocorticotropic hormone (ACTH) release from the pituitary and glucocorticoid secretion from the adrenal glands, reducing peripheral inflammation via the HPA axis (Mikkelsen et al., 1985). Second, the cholinergic anti-inflammatory pathway (CAP) involves the VN efferents: vagal ACh release activates the alpha-7 nicotinic ACh receptors (α7nAChR) on macrophages, suppressing the production of TNF-α, IL-1β, IL-6, and IL-18 (Biasi et al., 2013; Bonaz et al., 2018; Mantovani et al., 2011; Rubio et al., 2014). Although the VN does not directly innervate gut macrophages, it modulates them indirectly via enteric neurons expressing neuronal nitric oxide synthase (nNOS), vasoactive intestinal peptide (VIP), and choline acetyltransferase (ChAT), located near the α7nAChR-expressing macrophages in the gut’s muscular layer (Flint et al., 2012; Rubio et al., 2014).

Given its central role in regulating neuroimmune communication through the cholinergic anti-inflammatory pathway, VNS has primarily been investigated as an immunomodulatory therapy for CD. Because vagal dysfunction has also been implicated in depression, several studies have secondarily evaluated psychiatric outcomes (Borovikova et al., 2000; Sinniger et al., 2020; Wang et al., 2003). Sinniger et al. (2020) conducted a 6-month pilot study of VNS in patients with mild-to-moderate ileocolonic CD (diagnosed within 3 months before screening). Inclusion criteria included elevated C-reactive protein (CRP > 5 mg/L), fecal calprotectin (FC > 100 μg/g), and Crohn’s Disease Endoscopic Index of Severity (CDEIS ≥ 7). VNS devices were surgically implanted and titrated from 0.25 mA (30 s ON, 5 min OFF, pulse width 500 μs, f = 100 Hz) to 1.25 mA over 6 months. Vagal tone was assessed using Heart Rate Variability (HRV) at baseline and follow-up, with low-frequency (LF) and high-frequency (HF) components used to estimate sympathetic/parasympathetic balance. Plasma levels of IL-6, IL-23, and IL-12. TNF-α, TGF β-1, IFN-γ, and IL-10 were recorded, along with gut TNF-α and MIP-1α during routine gut biopsies. Patients taking previous anti-inflammatories were excluded.

After 12 months, VNS was associated with promising improvements in inflammatory endpoints: pro-inflammatory cytokines (IL-6, IL-12, IL-1β, IL-23, IL-17, TNF-α, IFN-γ, and MIP-1α) decreased, while anti-inflammatory cytokines (TGF-β1 and IL-10) increased in several patients. Gut biopsy samples demonstrated a 16-fold reduction in TNF-α and a 50% reduction in MIP-1α median levels. CRP normalized in four patients and declined in three others, while FC decreased in three of seven patients with elevated baseline values.

Overall, the evidence does not support it as an effective treatment for depression or anxiety in CD patients. Using the Hospital Anxiety and Depression Scale (HADS), neither depression nor anxiety scores changed significantly at 6- or 12-month follow-up, although patients reported reduced abdominal pain. These results align with earlier pilot studies (Bonaz et al., 2018; Sinniger et al., 2020), which suggested that while VNS can reduce inflammatory cytokine production and improve some disease activity markers, its effects on mood and anxiety remain insignificant.

Limitations of these studies include small sample sizes, lack of metabolic profiling, and the absence of long-term follow-up. Despite these limitations, VNS remains under investigation as an immunomodulatory therapy in CD, with preliminary evidence suggesting effects on inflammatory activity and gut-brain communication (Wang et al., 2003). Importantly, current evidence does not support VNS as an effective treatment for depression or anxiety in patients with CD.

### 5.3 Natural remedies

In addition to pharmacological and neuromodulatory approaches, several natural compounds emerged as potential therapeutic strategies; however, current evidence is predominantly preclinical, derived from murine models, in-vitro experiments, and engineered human 3-D EpiIntestinal tissue, with limited translational or clinical evidence in patients with Crohn’s disease (CD).

#### 5.3.1 Psilocybin

Recent research highlights the anti-inflammatory potential of psychedelic mushroom extracts. In-vitro studies have demonstrated reductions of nitrosative stress, inflammatory cytokines, and prostaglandins in lipopolysaccharide (LPS)-activated mouse and human macrophages. Psilocybin, the primary psychoactive compound, is metabolized to psilocin, which binds to serotonin 5-HT2A receptors, modulating immune function and cytokine production (Nkadimeng et al., 2020; Tylš et al., 2014). This biased 5-HT2A activation may engage β-arrestin-2 signaling, a negative regulator of nuclear factor-κB (NF-κB), thereby further suppressing inflammation (Cheshmehkani et al., 2017; Sharma and Parameswaran, 2015). Additional evidence suggests involvement of glucocorticoid signaling pathways (Robinson et al., 2023).

In human 3-D EpiIntestinal tissue models, psilocybin reduced multiple pro-inflammatory cytokines, including TNF-α, IFN-γ, IL-6, IL-8, MCP-1, and GM-CSF. These findings support the potential of psilocybin or related compounds to modulate the gut-brain axis (Nkadimeng et al., 2020). However, these results are limited to preclinical models. *In vivo* animal studies and human trials are required to establish efficacy and safety in CD populations (Robinson et al., 2023).

#### 5.3.2 *Rosemarinus officinalis* L. (rosemary)

*Rosmarinus officinalis* L., a medicinal herb rich in polyphenols (phenolic acids, diterpenes, and flavonoids), exhibits antioxidant, anti-inflammatory, and neuroprotective properties in animal models. Zhang and Lu (2025) investigated whether the anti-inflammatory properties of *Rosmarinus officinalis* L. could be utilized to treat depression in IBD patients. The polyphenols inhibited inflammatory pathways and cytokines (NF-kB, NLRP3 inflammasomes, IL-1β, IL-6, and TNF-α, iNOS, IL-10, etc.), improved the concentration of neurotransmitters (5-HT and dopamine), and modulated intestinal microbiota composition and metabolite profiles, including polyphenol-derived microbial metabolites.

These findings were limited to murine models, and their translation to human CD populations remains untested. Clinical trials are necessary to confirm whether rosemary’s anti-inflammatory and neuroprotective effects are relevant in CD-associated depression.

#### 5.3.3 Jasmine tea (JT) extract

Zhou et al. (2025) evaluated jasmine tea extract (JT) in chronic unpredictable mild stress (CUMS) mouse models of depression. JT reduced depressive behaviors, lowered serum and colonic pro-inflammatory cytokine levels (IL-1β, IL-6, and TNF-α), and improved oxidative stress markers, including malondialdehyde (MDA), superoxide dismutase (SOD), and catalase (CAT). JT also increased brain-derived neurotrophic factor (BDNF) expression within the hippocampus, suggesting a neuroprotective effect.

Gut microbiota analysis revealed an increase in diversity and enrichment of beneficial bacteria (*Firmicutes, Romboutsia, Blautia*) alongside a reduction in harmful taxa (*Proteobacteria, Escherichia-Shigella*). JT also reversed metabolic disturbances across feces, colon, serum, and cortex, including disrupted amino acid, bile acid, and lipid metabolism, particularly by normalizing neuroprotective amino acids (NAAG), restoring tryptophan levels, and increasing short-chain fatty acid (SCFAs) production. These effects are likely mediated by JT’s key compounds, such as L-theanine and tea polyphenols. While these preclinical findings are encouraging, human trials are needed to determine therapeutic relevance in CD.

Collectively, psilocybin, rosemary, and jasmine tea extracts demonstrate anti-inflammatory, microbiota-modulating, and neuroprotective properties in preclinical models. However, none have been tested in controlled trials in Crohn’s disease. Future studies should clarify whether these compounds provide measurable benefits in CD-associated depression and whether they are safe for clinical use.

## 6 Discussion

Depression and anxiety are highly prevalent and clinically meaningful comorbidities in Crohn’s disease (CD), associated with reduced quality of life (QoL), increased hospitalization, and greater risk of surgery needs and surgical complications (Lichtenstein et al., 2018). Accumulating evidence indicates that depressive symptoms in CD arise not solely due to psychosocial disease burden, but from dysregulation of the gut-brain axis (GBA), involving immune activation, epithelial barrier disruption, microbial imbalances, and altered neuroimmune signaling (Corridoni et al., 2014; Emge et al., 2016; Mikocka-Walus et al., 2016). Notably, depression frequently predates the onset of inflammatory bowel disease (IBD), including CD, emphasizing that there is a bidirectional and mechanistic relationship between the mind and the gut (Ananthakrishnan et al., 2013; Pennazio et al., 2015).

An important limitation of the current literature is that the strength of evidence varies considerably across therapeutic approaches. While biologic therapies have been evaluated primarily in clinical studies involving patients with Crohn’s disease or mixed inflammatory bowel disease (IBD) cohorts, many mechanistic pathways discussed in this review; including microbiota-derived metabolites, neuroimmune signaling, and several emerging therapeutic strategies such as ketamine and natural compounds; remain supported predominantly by preclinical or early-phase clinical studies. Consequently, extrapolation of these findings to patients with CD should be interpreted cautiously until validated in larger, disease-specific clinical populations (Cheshmehkani et al., 2017; Jones et al., 2023; Robinson et al., 2023; Sharma and Parameswaran, 2015; Wang et al., 2003; Zhang and Lu, 2025; Zhou et al., 2025).

Chronic intestinal inflammation in CD is characterized by elevated pro-inflammatory cytokines, including TNF-α, IL-6, IL-12, and IFN-γ, which may influence CNS function through neuroimmune, endocrine, and vagal signaling pathways (Corridoni et al., 2014; D’Onofrio et al., 2025; Mantovani et al., 2011; Sjöstedt et al., 2021; ten Hove et al., 2002; Yoo et al., 2020). Experimental evidence supports a role for inflammatory mediators in mood regulation, providing biological support for immune-to-brain communication within the gut-brain axis (Corridoni et al., 2014; Emge et al., 2016).

Breakdown of epithelial barrier integrity and microbial dysbiosis in CD promote systemic inflammation and neuroimmune signaling through translocation of microbial products and altered metabolite production (Cussotto et al., 2019; Graff et al., 2009; Kumar et al., 2020; Matteoli and Boeckxstaens, 2013; Santos et al., 1999). These changes have been associated with activation of pathways involving vagal signaling, stress responsiveness, and central immune regulation, providing a plausible mechanistic link between intestinal pathology and depressive symptoms (Corridoni et al., 2014; Cussotto et al., 2019; Emge et al., 2016; Graff et al., 2009; Kumar et al., 2020). However, direct clinical evidence linking specific microbial alterations to depression remains limited, and current models should therefore be interpreted as biologically plausible rather than definitively causal.

Biologic therapies targeting TNF-α reliably reduce intestinal inflammation and induce mucosal healing in CD (Di Petrillo et al., 2025; Gomollón et al., 2017). Several studies report modest improvements in mood and sleep following anti-TNF-α therapies, particularly in patients experiencing elevated inflammatory markers at baseline, such as C-reactive protein (CRP) (Feagan et al., 2013; Stevens et al., 2017). However, psychiatric outcomes remain inconsistent across trials, with some studies reporting no significant improvement in depression or anxiety scores despite biochemical and endoscopic remission (D’Onofrio et al., 2025). These findings suggest that while inflammation contributes to depressive symptoms, suppression of intestinal inflammation alone is insufficient to normalize neuropsychiatric outcomes in many patients.

Gut-selective biologics such as vedolizumab further highlight this distinction. Vedolizumab improves intestinal inflammation without systemic immune suppression (Stevens et al., 2017). However, its effects on mood and sleep apnea appear comparable to those of systemic biologics. This observation suggests that improvement in peripheral disease activity may indirectly influence GBA signaling, but does not conclusively establish direct CNS effects.

Serotonin (5-HT) is a critical mediator of gut-brain communication, with the majority synthesized by enterochromaffin cells in the gastrointestinal tract (Bercik et al., 2011; Gisbert and Chaparro, 2020; Kalliolias and Ivashkiv, 2016). Enteric serotonin regulates motility, secretion, and immune activity, and indirectly influences CNS serotonergic signaling through immune mediators and vagal pathways (Emge et al., 2016). While peripheral serotonin does not cross the blood-brain barrier, dysregulation of enteric 5-HT signaling may nonetheless impact mood through these indirect mechanisms.

Selective serotonin reuptake inhibitors (SSRIs) are commonly prescribed for depression in CD. Clinical studies summarized in this review demonstrate modest improvements in depressive symptoms and quality of life (QoL), with variable effects on gastrointestinal outcomes (Gracie et al., 2018). Conversely, observational studies associate SSRI use with increased corticosteroid dependence and disease activity in some patient populations (Späth-Schwalbe et al., 1998). These mixed findings may reflect the pleiotropic effects of SSRIs on immune function, gut motility, and microbial composition (Berman et al., 2000; Hunt et al., 2020; Kakazu et al., 1999; Mikocka-Walus et al., 2016). Collectively, serotonergic therapies appear to influence multiple components of the GBA, contributing to heterogeneous clinical responses.

Interpretation of psychiatric outcomes in Crohn’s disease may also be influenced by treatment-related factors. Biologic therapies may contribute to improvements in psychological wellbeing alongside reductions in inflammatory disease activity, making it difficult to determine whether improvements in mood reflect direct effects on gut-brain axis signaling or occur secondary to improved disease control (D’Onofrio et al., 2025; Stevens et al., 2017). This potential relationship highlights the importance of simultaneously evaluating psychiatric outcomes and objective measures of disease activity when assessing therapeutic effects.

Psychological interventions, particularly cognitive behavioral therapy (CBT), consistently improve depressive symptoms and QoL in CD, although effects on objective markers of intestinal inflammation remain limited (Hunt et al., 2020; Wang et al., 2023). The benefits of CBT are likely mediated through reductions in stress-induced sympathetic activation and improved coping strategies rather than direct modulation of mucosal immunity.

Neuromodulatory approaches such as vagus nerve stimulation (VNS) further support the role of neural pathways in immune regulation. VNS activates the cholinergic anti-inflammatory pathway and reduces pro-inflammatory cytokine production in experimental models and early clinical studies. In CD patients, VNS improves inflammatory markers and abdominal pain but does not consistently reduce depression or anxiety scores (Sinniger et al., 2020). These findings suggest that vagal anti-inflammatory signaling alone may be insufficient to reverse established depressive symptoms, underscoring the multifactorial nature of GBA dysfunction in CD.

Rapid-acting antidepressants such as S-ketamine demonstrate antidepressant and analgesic effects in CD patients undergoing essential surgical procedures, independent of changes in inflammatory markers such as IL-6 or C-reactive protein (Zhang et al., 2023). This dissociation indicates that ketamine’s effects on mood may be mediated primarily through central synaptic plasticity, rather than peripheral immune modulation; a contrast to the previously reviewed biologic or vagal approaches. However, due to small sample sizes, this association must be reviewed further.

Natural compounds discussed in this review, including psilocybin-containing preparations and plant-derived polyphenols, demonstrate anti-inflammatory, antioxidant, and neuromodulatory effects in preclinical models (Cheshmehkani et al., 2017; Jones et al., 2023; Nkadimeng et al., 2020; Robinson et al., 2023; Sharma and Parameswaran, 2015; Tylš et al., 2014; Zhang and Lu, 2025; Zhou et al., 2025). However, all evidence supporting their relevance to CD-associated depression remains preclinical, and clinical translation has yet to be established.

## 7 Future directions

Overall, depression in Crohn’s disease emerges from a complex and interconnected gut-brain axis involving immune activation, epithelial barrier dysfunction, microbial alterations, and disrupted neuroimmune signaling. The evidence synthesized in this review demonstrates that therapeutic strategies targeting individual components of this axis, including biologic anti-inflammatory agents, antidepressants, behavioral interventions, neuromodulatory approaches, and emerging pharmacological or natural compounds, produce heterogeneous and often incomplete improvements in depressive symptoms. This variability highlights the limitation of evaluating therapies in isolation and emphasizes the need for integrative, comparative approaches.

To advance mechanistic understanding and identify effective therapeutic strategies, future research would benefit from a comparative translational framework in which major therapeutic modalities discussed in this review are systematically evaluated. In preclinical contexts, such a study could utilize established models of intestinal inflammation alongside validated behavioral paradigms for depressive-like phenotypes, allowing simultaneous evaluation of immune, microbial, neural, and behavioral outcomes across intervention groups. This design would enable the direct comparison of distinct therapeutic strategies, including anti-inflammatory biologics, SSRIs, vagal neuromodulation, ketamine, and microbiota-targeting plant compounds, and clarify how each therapy influences distinct pathways within the GBA.

Importantly, these insights gained from comparative preclinical analysis should inform prospective clinical studies employing standardized gastrointestinal, psychiatric, and biological endpoints. This approach could facilitate identification of mechanism-specific and patient-specific treatment responses, clarifying which therapeutic strategies are most effective for particular inflammatory, microbial, or neuropsychiatric profiles. This approach can ultimately lead to a more rational selection of depression treatments for Crohn’s disease.

A precision-medicine approach may be particularly relevant to CD-associated depression given the heterogeneity of both intestinal disease and psychiatric manifestations. In IBD, integration of microbiome, metabolomic, transcriptomic, and other multi-omics data is increasingly being investigated to identify disease-specific biomarkers, improve patient stratification, and predict treatment response. Recent multi-omics studies have identified CD-specific microbial and metabolic signatures, although their clinical utility requires further validation (Bourgonje et al., 2023; Serrano-Gómez et al., 2025). Similar approaches in depression have identified alterations in microbial taxa and microbiota-associated metabolites, but substantial heterogeneity across studies currently limits the identification of a reproducible microbial signature of depression (Gao et al., 2023; Zhao et al., 2026). Future longitudinal studies should investigate whether combinations of microbial, inflammatory, metabolic, and psychiatric markers can identify distinct gut-brain axis phenotypes associated with depression severity or treatment response in patients with CD. Machine-learning and artificial intelligence (AI) approaches may eventually facilitate integration of these multidimensional datasets to support individualized prediction of disease trajectories and therapeutic responses; however, these strategies remain investigational and require prospective clinical validation (Ceccato et al., 2025).

Future studies should incorporate standardized objective disease activity measures, including C-reactive protein (CRP), fecal calprotectin, endoscopic healing indices, Harvey-Bradshaw Index (HBI), and Crohn’s Disease Activity Index (CDAI), alongside validated psychiatric outcome measures such as the Patient Health Questionnaire-9 (PHQ-9), Hospital Anxiety and Depression Scale (HADS), Hamilton Depression Rating Scale (HAM-D), Montgomery-Åsberg Depression Rating Scale (MADRS), and suicidality screening tools. Simultaneous assessment of inflammatory and psychiatric outcomes may help determine whether improvements in mood occur independently of disease control or arise secondary to reductions in disease burden.

A potential clinical framework could incorporate routine assessment of depressive symptoms alongside objective CD disease activity, followed by multidisciplinary evaluation for patients with persistent or clinically significant psychiatric symptoms. Integration of gastroenterology, primary care, psychology, and psychiatry may help distinguish depressive symptoms associated with active inflammatory disease from persistent depression requiring targeted psychiatric treatment. Future prospective studies should evaluate whether treatment pathways integrating psychiatric symptom severity with objective measures of CD activity improve both gastrointestinal and mental health outcomes.

In conclusion, a coordinated comparative strategy spanning both preclinical and clinical models represents a critical next step in better understanding the primary drivers of depression in Crohn’s disease. By directly contrasting therapeutic modalities across shared mechanistic and clinical outcomes, future studies can move beyond symptom management and toward more targeted interventions that address the multifactorial nature of gut-brain axis dysbiosis.
